# Segmented filamentous bacteria impede rotavirus infection via retinoic acid receptor-mediated signaling

**DOI:** 10.1080/19490976.2023.2174407

**Published:** 2023-02-05

**Authors:** Vu L. Ngo, Zhenda Shi, Baoming Jiang, Andrew T. Gewirtz

**Affiliations:** a Center for Inflammation, Immunity and Infection, Institute for Biomedical Sciences, Georgia State University, Atlanta, GA, US; b Division of Viral Diseases, Centers for Disease Control and Prevention, Atlanta, GA, USA

**Keywords:** Retinoic acid receptor, nos2, enterocyte migration

## Abstract

Prevention of rotavirus (RV) infection by gut-resident segmented filamentous bacteria (SFB) is an example of the influence of gut microbiota composition on enteric viral infection. Yet, the mechanism by which SFB prevents RV infection is poorly understood. A recent report that SFB colonization of germfree mice generates retinoic acid (RA) thus activating RA receptor (RAR) signaling, which protected against Citrobacter rodentium infection, prompted us to investigate whether this pathway might contribute to SFB’s protection against RV infection. Colonization of conventional mice by SFB indeed increased intestinal RA levels and direct administration of RA partially mimicked the protection against RV infection conferred by SFB. Moreover, blockade of RAR signaling eliminated SFB’s protection against RV infection. Blockade of RAR signaling did not impact RV infection in the absence of SFB, nor did it alter the protection against RV infection conferred by bacterial flagellin, which in contrast to SFB, is dependent upon IL-22 signaling. SFB/RA-mediated prevention of RV infection was associated with an RA-dependent increase in enterocyte migration, consistent with the notion that enhanced anoikis is the ultimate means by which SFB, IL-22, and RA impede RV infection.

## Introduction

The extent to which rotavirus (RV) infects individual hosts is highly heterogenous both between and within populations. Such heterogeneity is a determinant of whether exposure to a pathogenic RV strain results in disease as well as whether administration of rotavirus vaccines, which are live attenuated rotaviruses, will infect its host at levels sufficient to elicit protective adaptive immune responses. It has been suggested that one factor influencing heterogeneity of RV infectivity is gut microbiota composition.^1,2^ In accord with this notion, we previously reported that some colonies of mice harbor microbiota that conferred resistance to RV infection.^3^ Such RV-resistance associated with carriage of segmented filamentous bacteria (SFB), which when isolated and transplanted to new host mice conferred RV resistance, thus defining one discrete example of microbiota composition influencing proneness to viral infection. However, the mechanism by which SFB impeded RV was not well defined. We proposed a mechanism whereby SFB drives enterocyte proliferation and migration, thus leading to increased shedding and anoikis of villus tip epithelial cells, which are the primary site of RV infection, thereby reducing levels of replicating RV. The basis for proposing this mechanism came from our studies of bacterial flagellin, which promotes enterocyte migration and anoikis, and prevents RV infection via induction of IL-22 expression, which also drives enterocyte migration and anoikis.^4^ In contrast to flagellin, SFB’s protection against RV infection is independent of IL-22 but is associated with enterocyte proliferation/migration. Yet, whether increased enterocyte migration contributed to SFB’s protection against RV infection and, moreover, how SFB might impact this parameter remained unresolved.

Recent pioneering studies by Alenghat and colleagues found that SFB colonization protected germ-free mice against *Citrobacter rodentium* (*C. rodentium*) infection and revealed the underlying mechanism. Specifically, they found that SFB generated retinoic acid (RA), which activated host RA receptor (RAR) signaling, resulting in the upregulation of an array of genes, including inducible nitric oxide synthase (nos2), which was required for SFB’s protection against *C. rodentium*.^5^ Such findings prompted us to reactivate our investigation into mechanisms underlying SFB’s protection against RV. While SFB protects against RV in both germfree and conventional mice,^3^ we chose to work in the latter model as we view it to be more physiologically relevant. We found that SFB’s anti-RV action was independent of nos2 but indeed relied upon activation of RAR signaling, which was necessary and sufficient for SFB’s protection against RV infection.

## Methods

### Mice

The following mice were purchased from The Jackson Laboratory: C57BL/6, B6.129P2-Nos2tm1Lau/J (*nos2^−/−^*). Unless otherwise stated, mice were used at four weeks of age, and experiments were carried out using age- and sex-matched groups. Mice in all experiments were housed in an animal biosafety level 2 facility. Animal studies were approved by the Institutional Animal Care and Use Committee of Georgia State University.

### Acute rotavirus infection

Aged- and sex-match adult mice were orally inoculated with 10^5^ SD of murine rotavirus EC strain in 200ul PBS. Feces were collected for 12 consecutive days, and ELISA measured RV fecal antigens as previously described.^3,6^

### RV-Antigen/antibodies specific ELISA

Enzyme-linked immunosorbent assay (ELISA) was used to detect rotavirus antigens in mouse feces, as previously described.^3,6^ Briefly, 96 well EIA/RIA plates (Costar, 3590) were coated with Rabbit Anti-rotavirus Group-A (Biorad, AHP1360) capture antibody overnight at room temperature and blocked with 200ul of 1% BSA. Mouse fecal homogenates were prepared at 100 mg/mL concentration and were centrifuged to remove all debris. After blocking, supernatants of the homogenates were then incubated in the blocked plates. Stock murine rotavirus was used as a control. Hyperimmune Guinea pig anti-RRV were diluted at 1:1000 in 1% BSA and used as detection antibody. Followed by the incubation of HRP Donkey Anti-Guinea Pig antibody (Jackson ImmunoResearch, cat#706-035-148) secondary antibody at 1:5000 dilution in 1% BSA. All incubation steps after capture antibody were at 1 h at room temperature. TMB ELISA Substrate Solution (Invitrogen, cat#00420156) was utilized to develop the signal. TMB stop solution (KPL, cat# 50–85-04) was added after 5 minutes of TMB incubation. OD readings were taken at 450 nm.

### Serum/Fecal specific RV-antibodies ELISA

Serum and fecal anti-RV IgA and/or IgG were detected as previously described.^3,6^ Briefly, plates were coated with Rabbit Anti-rotavirus Group-A (Biorad, AHP1360) capture antibody overnight, followed by incubation RV, followed by incubation with serum or fecal superntants. The antibody amount was quantified following incubation with a horseradish-peroxidase-conjugated goat anti-mouse IgA or IgG diluted in a buffer containing 1% BSA.

### Mono-associated SFB transplantation

Donor fecal samples were collected from mono-associated SFB mice,^3^ suspended in 20% glycerol/PBS solution at 40 mg/mL, aliquoted, and stored in −80^O^C. Frozen mono-associated SFB suspensions were orally inoculated to the recipient mice in 200 µl volume.

### Administration of retinoic acid, RAR inhibitor (BMS493), and Aldh1a2 inhibitor (WIN18446)

Conventional mice were orally administered with 300ug all-trans retinoic acid (Sigma Aldrich cat# R2625) or DMSO (Sigma Aldrich cat# D8418) in 200ul corn oil every day for seven days before and during rotavirus infection.^5^ For RAR inhibitor (Torcis Bioscience cat# 3509) and Aldh1a2 inhibitor (Cayman Chemical cat# 14,018), 400ug BMS493 and WIN18446 suspended in 10% DMSO/corn oil was orally administrated in 200ul every other day for seven days before and during rotavirus infection.

### Flagellin administration

As previously described,^6^ flagellin was purified from flagella isolate from Salmonella Typhimurium via HPLC. Mice were intraperitoneally 10ug of flagellin every other day throughout the experiment.

### BrdU staining

As previously described,^4^ mice were intraperitoneally administrated with a 5-Bromo-2-deoxyuridine (BrdU) antibody (50ug of BrdU/g). Twenty-four hours post-injection, ileum of mice were harvested and embedded in OCT (Sakura, cat#4583). The tissues were sectioned into a 5um section and fixed with 4% formaldehyde for 30 minutes at room temperature. DNA denaturation was performed by incubating in 1.5 N HCl for 30 min at 37°C and rinsing three times with PBS. Slides were blocked with rabbit serum (BioGenex, Fremont, CA) for one h at room temperature, then incubated with anti-BrdU (Abcam) 2 hours at 37°C, and stained with 4’, 6-diamidino-2-phenylindole (DAPI). Slides were visualized by fluorescence microscopy. Villi, crypts, and epithelial cell migration were quantified using ImageJ.Intestinal epithelial cells isolation.

### Intestinal IECs isolation

IECs were isolated from ileum of small intestine by shaking tissues at 250rpm in HBSS media containing 1 mM EDTA and 10%FBS for 20 minutes.

## Retinoic acid quantification

IECs and intestinal contents were homogenized in PBS and RA level were measured using retinoic acid ELISA kit (MyBiosource, MBS706971) following manufacturer’s protocol. Briefly, samples were incubated with 50ul HRP-conjugated antibody at 37C for 40 minutes then the reaction was quenched, and absorbance was read using a micro-plate reader at 4OD50nm.

## Statistical analysis

All statistical analyses were done with GraphPad Prism, version 9.0. One-way ANOVA or student *t*-test.

## Results

**Figure 1.**
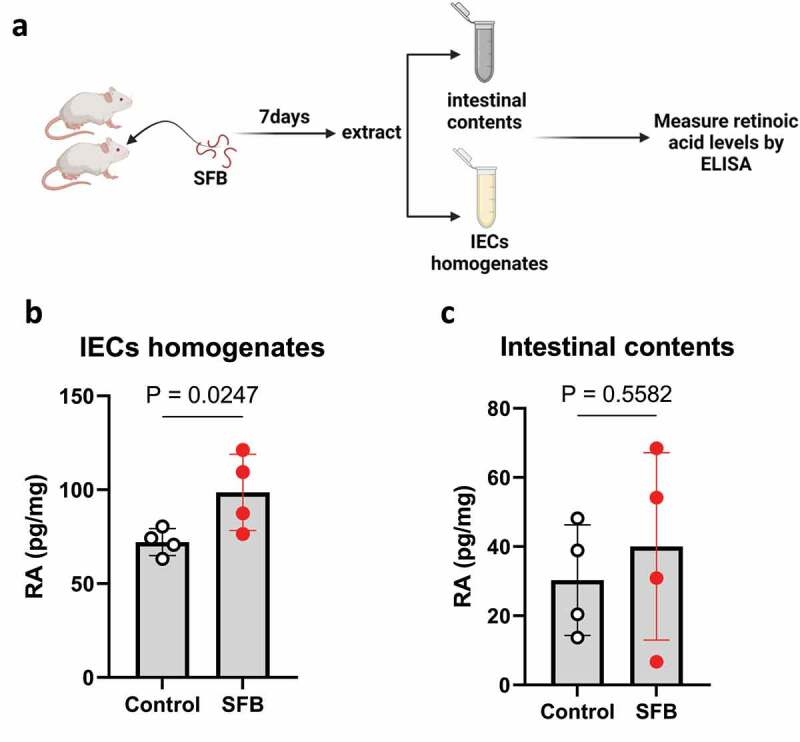
**SFB enhances RA level in host’s intestine**. Conventional C57BL6 were orally gavaged PBS or SFB. (a) Experiment approach. (b) ELISA measure of RA level of intestinal contents, and IECs homogenates. Results are mean ± SD of each group containing 4 mice.

Colonization of germfree mice with SFB increases RA levels.^5^ We hypothesized this observation would extend to conventionally colonized mice although one can imagine numerous ways that the vast array of endogenous microbes might produce, metabolize, and/or alter host production of RA. Hence, conventionally housed C57 BL/6 mice, purchased from Jackson Labs and verified by us to lack endogenous SFB, herein referred to as SFB^−^ mice, were colonized with SFB via oral gavage of feces from SFB-monoassociated mice (SFB-MA). Mice were euthanized 7 days later, at which time levels of intestinal RA were measured in the small intestine (Figure 1a), since this is the site of RV infection. We observed a statistically significant increase in RA levels of intestinal epithelial cell homogenates (Figure 1b) and a modest trend of elevated luminal RA levels that was not statistically significant (Figure 1c). These results accorded with the notion that SFB’s activation of RAR signaling might extend to conventionally colonized mice and thus prompted us to consider if RAR signaling might contribute to SFB’s protection against RV infection.

**Figure 2.**
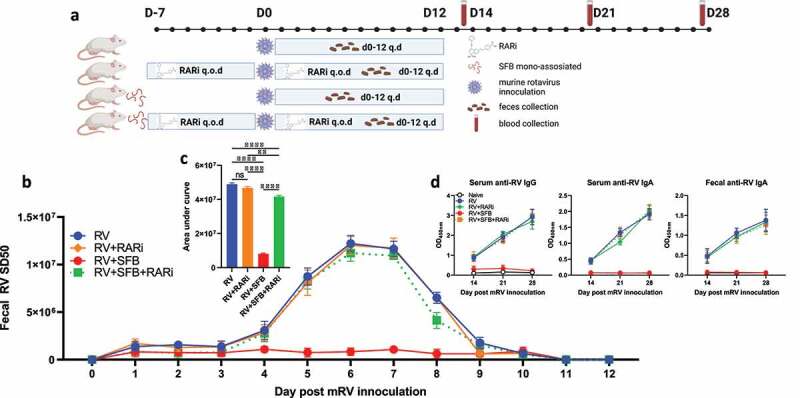
**SFB’s inhibition of mRV infection requires RAR signaling**. Conventional C57BL6 mice were orally administered PBS or SFB 7 days before rotavirus inoculation. RAR inhibitor (RARi BSM493, 400ug) or vehicle (10% DMSO in corn oil) was given orally to mice every other day (q.o.d) for 7 days pre- and post-inoculation. (a) Experiment approach. (b) ELISA measure of fecal rotavirus shedding levels over time, normalized to sample weight. (c) Areas under the curve analysis of **(b)**. (d) ELISA measure of rotavirus-specific antibodies in serum and fecal samples. Results are mean ± SD. Data represent two independent experiments with four to five mice per group that yielded similar results. ** p < .01. **** p < .0001. ns, not significant.

In accord with our published work,^3^ administration of SFB-MA feces to SFB^−^ mice conferred strong resistance to the asymptomatic rotavirus infection that otherwise occurs in adult mice as evidenced by their minimal fecal levels of RV antigens relative to those administered vehicle (germ-free feces). To investigate the potential role of the RAR pathway, mice were subjected to pharmacologic RAR inhibition via the RAR-inverse agonist (RARi: BMS493) using dosing that blocks SFB’s protection against *C. rodentium*.^5^ Such RAR inhibition did not, by itself, have a discernible impact on RV infection but largely eliminated the protection against RV conferred by SFB colonization (Figure 2a-c). As a secondary means to assay RV infection and thus help verify that our interventions truly reflected changes in RV infection, we measured levels of serum and fecal anti-RV antibodies, which are known to be generated weeks following RV infection. Generation of such antibodies at 14, 21, and 28 d post RV-inoculation closely correlated with day 1–12 levels of fecal RV antigens (Figure 2d). In the absence of SFB, RAR inhibition did not impact fecal RV antigen levels nor generation of anti-RV antibodies. These results further support the notion that RAR signaling was not mediating RV infection per se but, rather, was critical for the protection against RV infection conferred by SFB (Figure 2a-d).

**Figure 3.**
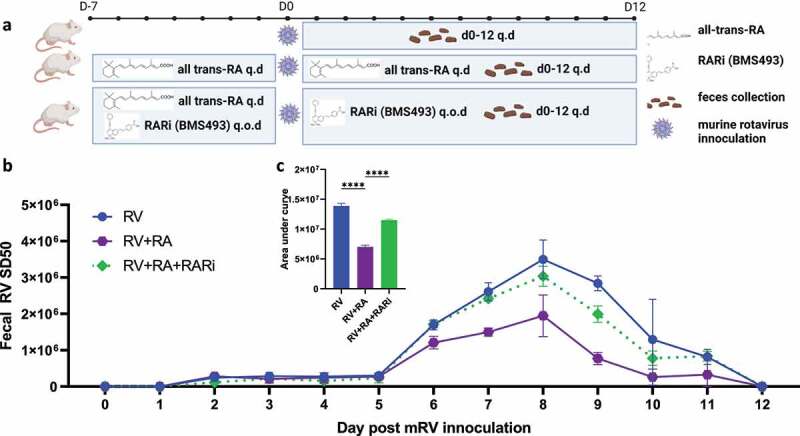
**Direct retinoic acid (RA) administration impeded RV infection in a RAR-dependent manner**. Conventional C57BL6 mice were orally administered all-trans retinoic acid (RA) 300ug or vehicle (10% DMSO in corn oil) every day (q.d.) for 7 days pre- and post-RV inoculation. (a) Experiment approach. (b) ELISA measure of fecal rotavirus shedding levels over time, normalized to sample weight. (c) Areas under the curve analysis of **(b)**. Results are mean ± SD. Data represent two independent experiments with four to five mice per group that yielded similar results. **** p < .0001.

**Figure 4.**
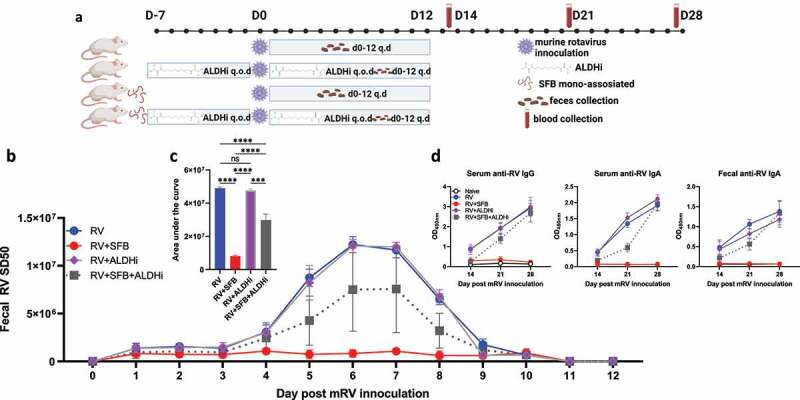
**Inhibition of host RA-production reduces SFB’s inhibition of RV infection**. Conventional C57BL6 mice were orally administered PBS or SFB 7 days before rotavirus inoculation. ALDH1A2 inhibitor (ALDHi WIN18446, 400ug) or vehicle (10% DMSO in corn oil) was given orally to mice every other day (q.o.d) for 7 days prior to and during rotavirus infection. (a) Experiment approach. (b) ELISA measure of fecal rotavirus shedding levels over time, normalized to sample weight. (c) Areas under the curve analysis of **(b)**. (d) ELISA measure of rotavirus-specific antibodies in serum and fecal samples. Results are mean ± SD. Data represent two independent experiments with four to five mice per group that yielded similar results. *** p < .001. **** p < .0001. ns, not significant. Note that data for the control groups (i.e. those without WIN18446) are same data as Figure 1 as these experiments were run In parallel to minimize numbers of animals needed.

We next examined the extent to which direct administration of RA could recapitulate the protection against RV infection conferred by SFB. Mice were administered RA or vehicle (10% DMSO in corn oil) via daily oral gavage following dosing previously shown to approximate luminal RA levels generated by SFB and, moreover, which impede *C. rodentium*. Such RA administration reduced fecal RV antigen shedding, albeit not to the extent seen with SFB. RA’s lowering of fecal RV antigen shedding was largely reversed by RARi inhibition (Figure 3a-c). These results strengthen the notion that activation of RAR contributes to SFB-mediated protection against RV infection. Total levels of RA, and thus RAR signaling, reflect RA produced by gut bacteria, including SFB, and host. Host production of RA is mediated by the Aldehyde Dehydrogenase 1 Family Member A2 (Aldh1a2), thus enabling a pharmacologic inhibitor of this enzyme, WIN18446, to impede host, but not SFB-mediated, RA generation.^5^ We found that WIN18446 did not, by itself, impact RV fecal antigen shedding but moderately reduced the stark reduction in this parameter conferred by SFB colonization (Figure 4a-c). Levels of RV antigen shedding were paralleled by levels of RV antibodies (Figure 4d). These results accord with roles for both host- and SFB-generated RA production in mediating RAR activation, which seemed critical for SFB-mediated protection against RV infection.

**Figure 5.**
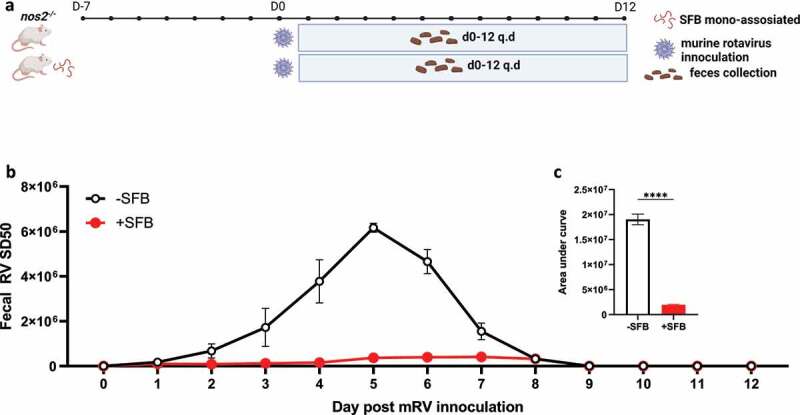
**SFB colonization inhibits mRV infection independent of nos2**. Conventional *nos2^−/−^* (nos2^−/−^) mice were inoculated or not with mono-associated SFB (40 mg/mL) seven-day prior to rotavirus inoculation. (a) Experiment approach. (b) ELISA measured fecal rotavirus shedding levels over time, normalized to sample weight. (c) Areas under the curve analysis of **(b)**. Results are mean ± SD, N = 5 mice per group **** p < .0001.

**Figure 6.**
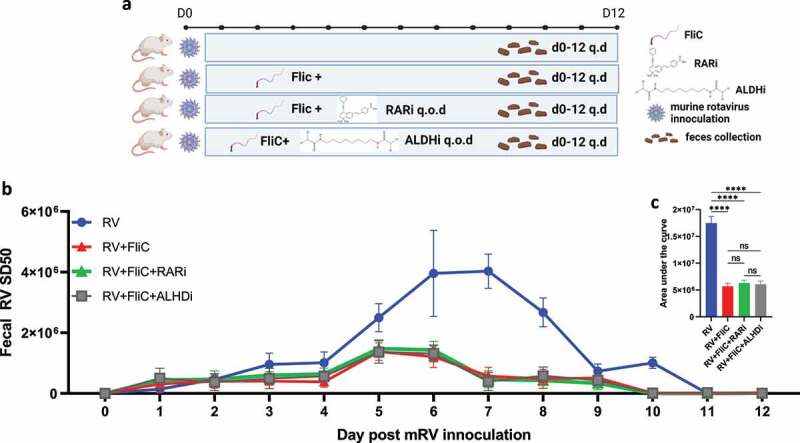
**Flagellin inhibition of mRV infection is independent of RAR signaling**. Mice were administered intraperitoneally with PBS, fliC (flagellin, 10ug) ± RARi or ALDHi every other day (q.o.d) during rotavirus infection. (a) Experiment approach. (b) ELISA measured fecal rotavirus shedding levels over time, normalized to sample weight. (c) Areas under the curve analysis of **(b)**. Results are mean ± SD. **** p < .0001. ns, not significant.

**Figure 7.**
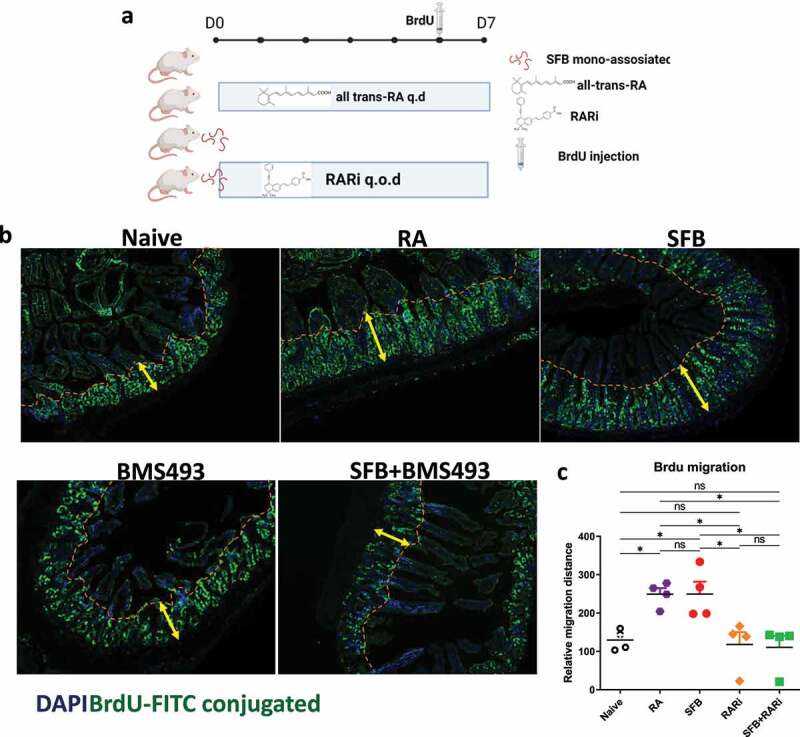
**SFB’s impact on RV infection associates with RAR-mediated enterocyte migration**. Conventional C57BL6 mice were administered PBS, SFB, or began daily oral RA treatment during which some mice were simultaneously subjected to RAR inhibition. 7 days later, BrdU was intraperitoneally injected. 24 hours later intestinal tissue was harvested. (a) Experiment approach. (b) Fluorescence microscopy. (c) BrdU migration distance. Data represent two independent experiments with four mice per group and yielded similar results. Results are mean ± SD, N = 4 * p < .1.

In considering how RAR activation might impede RV infection, we first investigated the role of inducible nitric oxide synthase (nos2), which is critical for SFB-mediated protection against *C. rodentium* as evidenced by complete loss of protection in nos2^−/−^ mice.^5^ In contrast, we observed that SFB’s protection against RV infection remained intact in such mice (Figure 5a-b). We next considered the extent to which RAR’s inhibition of RV infection might resemble that conferred by bacterial flagellin, which is known to protect against RV via eliciting production of IL-18 and IL-22. Neither flagellin, IL-18, nor IL-22 are required for SFB to impede RV infection.^3^ We envisioned a hypothesis capable of unifying these results, namely that activation of RAR signaling might be the ultimate means by which both flagellin and SFB protect against RV infection. However, flagellin’s protection against RV was not reduced by either WIN18446 or BMS493, arguing against this possibility but nonetheless providing further evidence of the specificity of these compounds (Figure 6a-c). We thus considered an alternative unifying hypothesis, namely that driving enterocyte migration up the crypt-villus axis is the ultimate means of preventing RV infection and that RAR signaling might mediate the IL-22-independent increase in enterocyte migration previously observed in SFB-colonized mice.^4^ Hence, we used bromodeoxyuridine (BrdU) labeling to measure the extent to which RAR signaling was sufficient and necessary for SFB-induced enterocyte migration. We observed that direct administration of RA enhanced enterocyte migration up the crypt-villus axis to a similar extent as SFB colonization. Furthermore, RAR inhibition did not significantly impact the basal rate of enterocyte migration but completely inhibited the SFB-induced increase in this parameter (Figure 7a-b). Collectively, our results suggest SFB’s prevention of RV infection results in large part from its activation of RAR signaling, which drives enterocyte migration, which increases extrusion of villus tip cells that are preferentially infected by RV.

## Discussion

Recent appreciation that segmented filamentous bacteria (SFB) and perhaps other select gut bacteria have the capacity to impede infection of enteric viruses, including rotavirus (RV,) has suggested the possibility of gut-microbiota-based strategies to understand heterogeneity of RV infection. Such heterogeneity may be germane to understanding both RV pathogenesis and efficacy of RV vaccines, which are live attenuated RV, which need to infect their hosts to elicit immunity. The extent to which SFB is, or is capable of being, a significant component of the human microbiotas is far from clear.^7,8^ Regardless, administering SFB or SFB-like bacteria to humans would not be broadly viable in that SFB, despite having many beneficial activities, increases proneness to an array of chronic diseases, especially those driven by Th17 cells and, moreover, is very hard to eliminate from an individual host.^9^ However, understanding the mechanism by which SFB protects against RV infection may yield harnessable strategies that could help manage the RV-induced disease burden. This report takes a concrete step toward this goal by identifying that the retinoic acid receptor (RAR) signaling pathway plays a critical role in linking SFB colonization to protection against RV infection.

That SFB colonization increases intestinal RA levels and, consequently, activates RAR signaling was recently discovered by Allenghat and colleagues.^5^ Their pioneering study demonstrated such RAR activation led to induction of nos2, which was critical for SFB to impede colonization of *C. rodentium* via use of a specific RAR inhibitor and nos2-deficient mice. That, in our hands, this same inhibitor did not impact RV-infection in SFB-free mice nor impede prevention of RV infection by flagellin but reversed SFB’s inhibition of RV infection supports the purported specificity of this pharmacologic agent and argues that the RAR pathway is central to SFB’s antiviral action. Allenghat *et al*.’s use of another specific inhibitor, namely an inhibitor of mammalian aldehyde dehydrogenase, which mediate generation of RA from retinol, combined with measuring intestinal RA levels, indicated that the increased RA they observed was generated by SFB rather than host dehydrogenases. In our hands, this inhibitor partially reduced the extent to which SFB’s prevented RV infection but did not impact RV in SFB-negative or flagellin-treated mice. This result is consistent with the possibility that, in contrast to the case for germ-free mice, which were used by Allenghat *et al*., in conventional mice, addition of SFB to a complex microbiota result in activation of RAR signaling via production of RA generated by both host and SFB dehydrogenases and/or that the pharmacologic properties of the inhibitor are distinct in conventional germ-free mice. Additional experimentation would be needed to investigate these possibilities.

**Figure 8.**
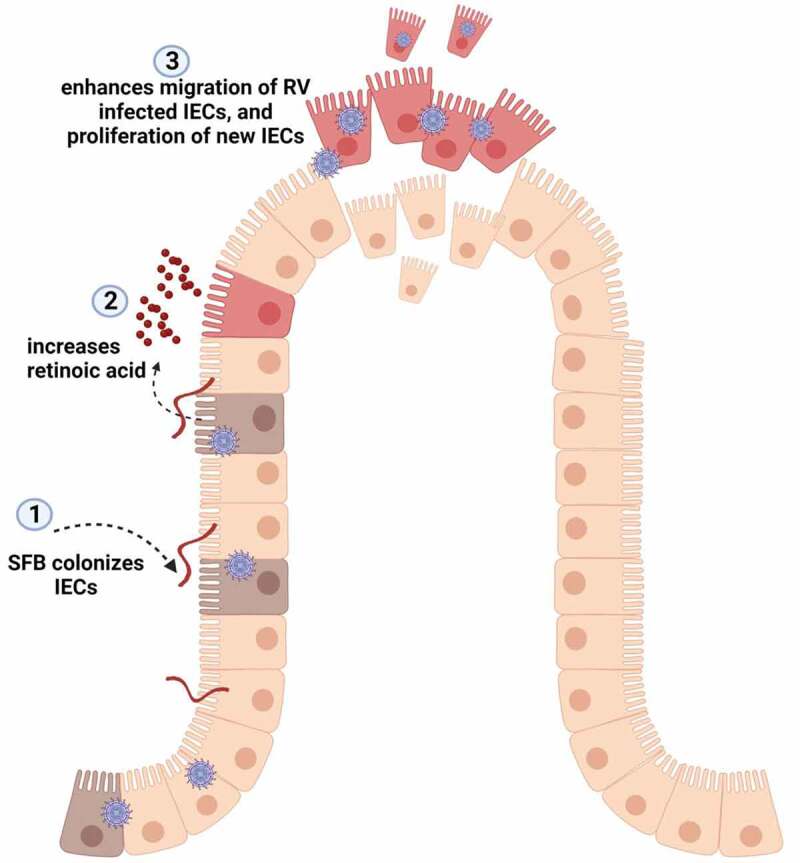
**Proposed model for SFB prevents rotavirus infection via RA-mediated signaling**. During rotavirus infection, SFB colonization of the ileum of the small intestine enhances level of retinoic acid, which results in accelerated migration/expulsion of RV-infected epithelial cells and proliferation of new IECs.

Another contrast between SFB’s prevention of *C. rodentium* vs. RV infection is that the latter was independent of nos2. This contrast is not particularly surprising in that nos2 is but one of many genes induced by RAR activation and is not thought to contribute to antiviral defense. In any case, it suggests another consequence of RAR signaling mediates SFB’s antiviral action. Work from Gomez et. Al, whom observed RA could attenuate RV infection in vitro, suggested a role for PPARγ signaling.^10^ Yet, neither SFB, flagellin nor IL-22 impacts RV infection in vitro, leading us to consider mechanisms that might only manifest in vivo. Our previous observations that the resistance to RV infection in mice administered flagellin, IL-22, and SFB associated with increased enterocyte migration, led us to hypothesize that this event may be a downstream consequence of a number of signaling pathways, including RAR.^6^ That RA administration drove enterocyte migration and reduced RV infectivity, combined with the observation that SFB induced enterocyte migration was RAR-dependent supports this hypothesis. As schemtized in Figure 8, such enhanced enterocyte migration results in increased extrusion of villus tip epithelial cells, i.e., anoikis, which we view as an orderly purposeful cell death that not only occurs without epithelial barrier disruption of inflammation but also likely results in destruction of intracellular viruses.^4^ Additionally, such increased epithelial cell turnover may reduce the abundance of the highly differentiated cells that are preferentially targeted by RV.

How RAR activation links to enhanced enterocyte migration is not yet clear. Indeed, RA has long been used as means of inducing cell differentiation while increased enterocyte migration is typically associated with greater levels of enterocyte proliferation and a reduced state of differentiation.^11^ Thus, further studies are needed but we submit that the intertwining of proliferation, migration, and differentiation is complex, tissue-specific, and far from clear. We anticipate that attaining better understanding of how RAR impacts enterocyte proliferation, migration, and/or differentiation, and how such processes may be modulated by nutritional factors, including the retinol precursor Vitamin A, may pave the way for practical, safe approaches to temporarily modulate RV infection. We envisage developing strategies to enhance enterocyte migration as a means of providing transient nonspecific protection of differentiated enterocytes from RV infection. Yet, using the converse approach in vaccination might be more practical. Specifically, we envision that transiently impeding enterocyte migration by blocking RAR signaling prior to administration of live attenuated RV vaccines may increase their infectivity, potentially overcoming the poor immunogenicity that associates with their modest efficacy in some low-income countries.
